# Relating the Biogenesis and Function of P Bodies in *Drosophila* to Human Disease

**DOI:** 10.3390/genes14091675

**Published:** 2023-08-24

**Authors:** Elise L. Wilby, Timothy T. Weil

**Affiliations:** Department of Zoology, University of Cambridge, Downing Street, Cambridge CB2 3EJ, UK; elw63@cam.ac.uk

**Keywords:** processing bodies, biomolecular condensates, translational regulation, *Drosophila*

## Abstract

*Drosophila* has been a premier model organism for over a century and many discoveries in flies have furthered our understanding of human disease. Flies have been successfully applied to many aspects of health-based research spanning from behavioural addiction, to dysplasia, to RNA dysregulation and protein misfolding. Recently, *Drosophila* tissues have been used to study biomolecular condensates and their role in multicellular systems. Identified in a wide range of plant and animal species, biomolecular condensates are dynamic, non-membrane-bound sub-compartments that have been observed and characterised in the cytoplasm and nuclei of many cell types. Condensate biology has exciting research prospects because of their diverse roles within cells, links to disease, and potential for therapeutics. In this review, we will discuss processing bodies (P bodies), a conserved biomolecular condensate, with a particular interest in how *Drosophila* can be applied to advance our understanding of condensate biogenesis and their role in disease.

## 1. Introduction

Biomolecular condensates, commonly thought to form by phase separation, are self-organising regions of the cytoplasm or nucleoplasm [1]. Having been observed in a myriad of cell types, condensates are thought to be involved in a wide variety of functions, including DNA replication [2], ribosome formation [3,4], and the post-transcriptional regulation of mRNA [5,6]. Processing bodies (P bodies) are an evolutionarily conserved condensate, first discovered in yeast [7] and subsequently detected in many species, including *Arabidopsis* [8,9], *Caenorhabditis elegans* [10,11,12], *Drosophila* [13,14], mice [15], and humans [16,17]. Lacking a surrounding membrane and composed primarily of RNAs and proteins, P bodies are a type of ribonucleoprotein (RNP) granule (note that the terms condensate and granule are often used interchangeably in the literature). Moreover, as hubs of RNA metabolism, P bodies have been defined by the presence of specific proteins associated with mRNA degradation and translational repression [7,13].

Work in yeast, mammalian cell lines (in cellulo), and in vitro systems have been foundational in our understanding of P bodies, detailing their composition [6,18], principles of assembly and disassembly [19,20], responses to environmental change [21,22], and potential roles in neurodegeneration [23], viral infection [24], and cancer pathogenesis [25]. The next step is to further understand the biological role of P bodies, and this will benefit from experiments using multicellular model systems that are amenable to genetic and physical manipulation, as well as providing a whole organism platform to assess the impact of a disease. *Drosophila* is particularly well suited to the study of P bodies in vivo (defined in this context as P bodies that exist within a complex cellular system, tissue, or organism) due to their conservation of key proteins and organ systems as well as their ability to model human disease.

## 2. Drosophila P bodies Have Complex Compositions and Multiple Functions and Are Present in Diverse Tissues

P bodies were first observed in cultured *Drosophila* Schneider 2 (S2) cells [13,26] and much of what we know relating to the formation, maintenance, and protein content of P bodies in flies comes from this system [13]. Whilst powerful, there are limitations to S2 cells as they behave differently depending on their lineage history and experimental context [27]. P bodies have since been identified and are being studied in a wide range of *Drosophila* cells and tissues to elucidate their function from the molecular to the organismal level.

In mammalian cultured stem cells, P bodies are generally thought to help balance between maintaining stemness and cell differentiation [28,29]. Similarly, in *Drosophila* intestinal stem cells, in vivo P bodies have been detected, but these were absent in the differentiated daughter enterocyte cells [30]. By repressing the formation of P bodies, pro-differentiation protein expression is elevated and a loss of the parent stem cells in the intestine is observed [30]. P bodies, in this context, are proposed to prevent differentiation whilst simultaneously keeping these cells primed for this transition.

In contrast, both the germinal stem cells as well as the differentiated cells in the *Drosophila* testes, the spermatogonia and spermatocytes, contain P bodies [27,31]. The DEAD box helicase maternal expression at 31B (Me31B), a conserved P body protein, has been shown to be critical for the translational repression of *nanos* mRNA, which prevents the de-differentiation of the spermatogonia. However, the link between the function of Me31B and P bodies has not been explicitly investigated [32]. This finding underlines an important paradox when considering P bodies, or any biomolecular condensate: is the observed role of a condensate protein based on the role of the protein itself or the role of the protein in the context of the condensate?

To date, much of the developmental and organismal-based P body data in *Drosophila* comes from studies in the egg chamber, containing the oocyte and nurse cells, and early embryo [14]. More broadly, decades of research have identified multiple populations of RNP granules in the oocyte, supporting nurse cells, and embryos which are known to have divergent functions, unique subcellular localisations, and overlapping yet distinct protein compositions [33]. For example, early observations in nurse cells identified ‘sponge bodies’ based on their electron microscopy morphology [34]. Whilst sponge bodies have been classified as discrete granules [35], they are increasingly grouped with the P bodies in the literature due to similarities in protein content and proposed function. This highlights one of the challenges as the field matures, which is to determine what gives a condensate its identity and how that identity is reflected in the naming convention.

### 2.1. Focus on: P Body Protein Content in Drosophila

Proteins make up a significant proportion of P bodies and this specific subsection will highlight our current understanding of those proteins in *Drosophila*. To elucidate P body biogenesis in *Drosophila*, it is critical to understand the entire protein complement of P bodies. While co-immunoprecipitation and interactome analysis of a key P body marker, Me31B, in the egg chamber [36,37] and embryo [36] have been completed, it is challenging to discern which of the proteins identified in these experiments are bona fide P body components. This is due to the promiscuous nature of Me31B; it is present in several different germ granules in the egg chamber [33] and has a significant presence in the dilute cytoplasmic phase [38]. Additionally, technical challenges have prevented the deciphering of the entire protein content of P bodies, which has previously been possible in cell culture by fluorescence-activated particle sorting followed by mass spectrometry [6]. Co-localisation studies in S2 cells and egg chambers have informed our current knowledge of the P body proteins in *Drosophila* (Table 1). However, this table likely underrepresents the grand total of proteins present in P bodies.

Below, we focus on some notable data from Table 1:

- Co-localisation studies in the female germline have shown unique RNA binding proteins present in these P bodies, such as Cup, Oo18 RNA binding protein (Orb), Squid (Sqd), Heterogeneous nuclear ribonucleoprotein at 27C (Hrb27C), Exuperantia (Exu), Ypsilon Schachtel (Yps), Lost, and Bruno 1 (Bru1) [35,40,42].
- The CCR4/Not complex, which is localised to P bodies in yeast, mammalian cells, and S2 cells [7,13,17], appears to localise to different cytoplasmic granules in the egg chamber [14].
- The miRNA machinery is localised to P bodies in S2 cells [13], but this is not the case in the egg chamber or embryos, the miRNA machinery present in GW bodies is separate from the P bodies [44].

Together, this highlights that the P body protein content is not necessarily consistent across cell and tissue types, and this presumably influences the recruitment and regulation of mRNAs.

**Figure 1:**
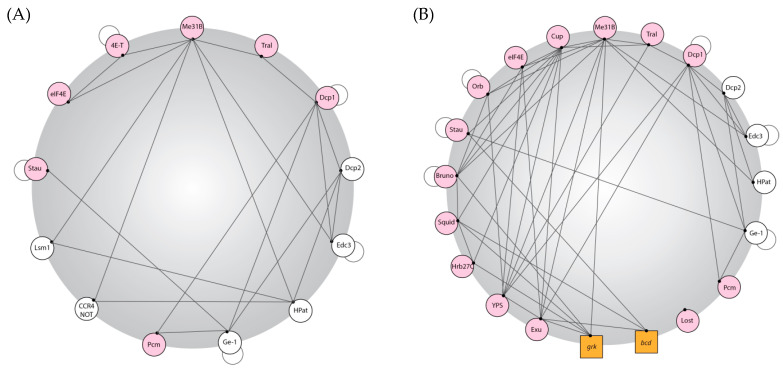
***Drosophila* P body protein interactions.** P body proteins in *Drosophila* have dense interaction networks coupled with the ability to bind RNA. Created using publicly available data on BioGRID and based on interaction maps produced for yeast and human P body proteomes [20]. (**A**,**B**) An interaction map of *Drosophila* P body proteins. Proteins are represented in circles and RNA is represented in squares; a line shows potential protein–protein or protein–RNA interactions (these interactions were elucidated using biochemistry; thus, the spatial information, whether this interaction is P-body-specific, is lost, so these diagrams represent maximal/potential interactions that could be taking place in a P body); a pink circle shows that the protein can bind RNA; and an adjoining circle denotes that the protein can self-bind. (**A**) Interactions between canonical P-body components in *Drosophila*; (**B**) interactions between P body components found in *Drosophila* egg chambers. To consider all possible factors, minimal stringency was applied to the existing data in the field.

For in vivo phase separation to occur, the protein content of a granule must be governed by certain rules including: (1) a network of interactions between the proteins must exist that are dense and redundant; (2) a significant proportion of the proteins must be able to bind to RNA specifically or non-specifically; (3) a large proportion of proteins must have low-complexity sequences or high levels of intrinsic disorder to allow for weak, non-specific interactions between proteins and RNA [20]. These three requirements are well met for known *Drosophila* P body proteins. Rules 1 and 2 are demonstrated for *Drosophila* P body proteins in Figure 1, and rule 3 was demonstrated by completing disorder predictions across the set of known P body proteins in egg chambers. Over 50% of the residues in the P body proteome are predicted to be disordered, and the fraction of disorder of proteins associated with the P body is over 99.9% more disordered than any possible random sized-matched set of proteins taken from the *Drosophila* proteome [38].

Importantly, there is significant tissue specificity in the protein complement of P bodies, and proteins could be recruited in a cell-dependent context to provide specific functions and likely provide additional contacts to increase the interaction density and RNA binding.

### 2.2. In Vivo P Bodies Can Undergo Regulated Changes

Patient biopsies have shown that P bodies within the same tissue type in humans can also exhibit variation [45], and the *Drosophila* egg chamber provides a system to test the complexity of such in vivo P bodies. Examples of this diversity include the stoichiometry of resident P body proteins that appears to differ between the oocyte and nurse cells despite these cells being interconnected via cytoplasmic bridges [35,40]. P body RNA content can also be different depending on the subcellular localisation within the oocyte [38,40,42]. Second, immunoelectron microscopy on ultra-thin frozen sections shows that oocyte P bodies have an ultrastructural organisation consisting of an inner core that is devoid of ribosomes and enriched with specific proteins and where non-translating mRNA is stored, and a periphery that is enriched with ribosomes, a translational activator, and an actively translating mRNA [42]. Third, *gurken* (*grk*) mRNA translation only occurs at P bodies located at the dorso-anterior corner of the oocyte [40]; this is thought to be due to the post-translational modification of translational activators in P bodies specifically at this sub-cellular location [46].

Developmental and environmental cues can also dramatically influence P body content, form, and function. In the early egg chamber, nurse cell P bodies have been shown to be rapidly and reversibly enlarged upon the addition of environmental stressors [47]. Similar observations have been reported in mammalian and yeast P bodies. Starvation cues originating in the brain of *Drosophila* drive post-translational modifications and the re-organisation of the microtubule network in egg chambers, which leads to the aggregation of the P bodies [48,49]. This change is hypothesised to allow for the reversible storage and protection of oocyte-specific RNAs until the environmental stress has passed [48].

In mature oocytes, *bicoid* (*bcd*) mRNA is localised to the oocyte anterior, where it is similarly stored and translationally repressed in stable P bodies. At this stage, the entirety of the *bcd* mRNA content is found in P bodies [38,42]; this is likely to ensure complete translational repression until protein expression is required in the embryo for axis patterning. This is in contrast with evidence from mammalian cells that shows of RNAs that localise to P bodies, individual species only have 15–30% of their transcripts in P bodies [50]. This suggests that one possible way that cells can ‘tune’ the expression levels of proteins is by associating different amounts of RNA with P bodies.

In some diseases associated with RNA dysregulation, RNA can become disproportionately segregated into aggregates [51]. There is growing therapeutic interest in the disassembly of these and other aggregates that coincide with pathogenesis. Interestingly, there is a time in *Drosophila* development when the stable co-localisation between RNA and P bodies dramatically changes, which could help us to understand the properties of condensate disassembly. Egg activation, a universal event that ensures that the oocyte is competent to be fertilised and begin embryogenesis [52], has been shown to result in the disassembly of stable P bodies [38,42,53]. Best supported by data from *bcd* mRNA, the events of egg activation are hypothesised to release repressed mRNAs that are stored in the core of the P body, thus ensuring the correct spatiotemporal translation [38].

Later, in early embryogenesis, smaller and more dynamic P bodies reform [38], and this offers a unique avenue for the study of de novo condensate formation. Current work shows degradation intermediates for short-lived mRNAs accumulating in reformed P bodies during embryogenesis [54], and P body components Me31B, Decapping protein 1 (DCP1), Staufen (Stau), and Pacman (Pcm) accumulate with *oskar* (*osk*) mRNA, and this correlates with the degradation of the mRNA [55]. Additionally, biochemical data suggest that Me31B transitions from a translational repressor to a beacon for mRNA degradation after the maternal to zygotic transition (MZT) [56]. Overall, the ability of Me31B protein and P bodies to change architecture and execute different functions depending on the cellular environment highlights an exciting aspect of condensate biology.

In mammalian and *Drosophila* neurones, differences in P body components are particularly evident [57,58]. A variety of granules exist, with most containing a limited subset of the canonical P body proteins and a range of specialised components [59,60]. The differences in these neuronal RNP granules have led to inconsistent nomenclature, where granules are sometimes termed P bodies and other times are not. This raises more general questions—what makes a granule a P body and what gives a biomolecular condensate its identity?

Nevertheless, these granules share some functional and material similarities to P bodies in oocytes and embryos and are similarly postulated to act as sites of translational repression until environmental cues lead to their disassembly [61,62]. Neuronal RNP granules also have been found to reform after dispersal [63], similar to P bodies in the early embryo. It is tempting to speculate that there are conserved principles regulating RNP granule assembly and disassembly between dissimilar cell types.

## 3. Current Understanding of the Requirements for Drosophila P Body Formation

Proteins are critical drivers of phase separation and P body formation. In vitro, protein properties, including valency [64,65], regions of disorder [66,67,68], sequences of low complexity [69,70,71], and the capacity to form weak, non-specific, temporary interactions, have been shown to be relevant to influence phase separation [71,72,73,74,75,76,77]. In mammalian cells, three key P body proteins that have some of these properties, DDX6 (Me31B), Lsm14A (Trailer hitch (Tral)), and eIF4E-Transporter (4E-T/Cup), were required for P body formation under all conditions tested [17,78,79,80], suggesting that these proteins act as scaffolds for P body assembly [19] (scaffolds are broadly defined as proteins and/or RNAs that function to concentrate condensate components [81]). Budding yeast appear more complicated, with Enhancer of decapping 3 (Edc3) and Sm-like protein 4 (LSm4) acting together to form the scaffold [82,83].

As discussed, *Drosophila* P bodies exist in multiple tissue types and often have specific protein contents. Disruption of P body proteins has revealed differences in the proteins required for P body formation between *Drosophila* tissues (Table 2).

Below, we highlight some noteworthy data from Table 2.

- HPat and Ge-1 are the only two proteins that appear to be required for the formation of P bodies in more than one cell type in flies [13,30,41]. These could potentially represent the ‘core assembly machinery’ for *Drosophila.*
- Me31B can act as a scaffold for phase separation in vitro [38], and loss of Me31B results in the disassembly of P bodies in S2 cells [13]. However, in *Drosophila* nurse cells, when Me31B was mutated, such that it was not able to self-aggregate or be recruited to condensates, Cup and Tral still formed condensates [84]. This suggests that Me31B is not specifically required for condensate formation in this scenario [30,84].
- Tral is not required for P body formation in S2 cells [13], but Tral knockdown leads to smaller P bodies in intestinal stem cells [30], their disassembly in nurse cells [81], and an altered morphology in the mature oocyte [38].
- Edc3 has an inconsistent role in P body formation, with knockdown of Edc3 showing no observable effect in S2 cells [13] but an increased size in intestinal stem cells’ P bodies [30].
- In S2 cells, all members of the miRNA machinery tested were shown to be necessary for P body formation [13], whereas reduction of these factors had no direct consequence on P bodies in intestinal stem cells [30].

It is worth considering why there could be such different outcomes in P body morphology when disrupting a specific protein. Firstly, there may be intrinsic differences in the cellular environment that drive condensate assembly and properties, including protein and RNA composition, concentrations, stoichiometries, binding capacity, and post-translational modifications. In addition, variance could arise from differences in the experimental conditions. These may include the visualisation of P bodies with different marker proteins, different methods used to alter the level of protein expression, and stress conditions introduced by the experimental setup. Additional experiments will be required to verify whether these observed inconsistencies are due to genuine cellular differences.

## 4. In Vivo Exploration of P Body Biogenesis

The *Drosophila* egg chamber offers an insightful model to test how phase separation functions in vivo, with many techniques readily available for use in *Drosophila* (highlighted in Section 4.1). This is illustrated by elegant work on the phase-separated condensates responsible for the transport and translational regulation of *osk* mRNA, a posterior determinant in *Drosophila* (note that similar techniques could be applied to *Drosophila* P bodies in the future). Historically, Bruno 1 (Bru 1) has been shown to bind to sites in the 3′ UTR of *osk* mRNA and mediate oligomer formation [91], which allows individual mRNPs to self-assemble into higher-order structures capable of moving many mRNAs in a single transport particle [91]. Recently, it was confirmed in vitro that Bru 1 can act as a scaffold for phase separation and that the N terminal domain is critical for this self-assembly process in vivo [92]. Consistent with predictions of what makes a protein a scaffold [93], Bru 1 is modular, with multiple RNA recognition motifs, regions of intrinsic disorder, and several low-complexity domains.

The *osk* transport particles, also exemplify how the physical nature of the phase-separated granule influences its biological function. These particles have been shown to be ‘solid-like’ and this is required to maintain the RNA within the granule. When the condensate properties were experimentally altered to be more ‘liquid-like’, *osk* mRNA was prematurely released from the transport particle, resulting in mis-localised translation [92]. Similar experiments in *Drosophila* would be useful to identify whether the physical nature of the P body is necessary for their biological function in other contexts.

Whilst often overlooked, RNA does appear to have a complex role in the biogenesis of biomolecular condensates [94]. RNA has been shown to be critical in the formation of P bodies in yeast and mammalian cell lines [7,16], and manipulation by RNase A or cycloheximide in *Drosophila* S2 cells and nurse cells shows that these P bodies are also highly dependent on RNA for their integrity [13,14]. Contrastingly, P bodies are more resistant to a reduction in RNA in oocytes and embryos [14,38,44], suggesting that protein–protein interactions are more important for the structures of these P bodies [38].

Unfortunately, there are only a few well-documented examples of RNAs that associate with P bodies in *Drosophila* [42,54,95], and more research is needed to fully understand the importance of RNA in P body biogenesis.

Possible ways to identify additional RNAs that are associated with P bodies include particle sorting followed by transcriptomics, which has been successfully implemented in mammalian cells [6] and the more common and lower-throughput approach of assessing co-localisation between P body proteins and RNAs by single-molecule fluorescence in situ hybridisation (smFISH). To find candidates for co-localisation studies, RNAs can be selected based on a low translational status and GC content, particularly in the 3′ UTR [50]. Of note, *bcd* mRNA when stored in P bodies fits both criteria.

Once RNAs that localise to P bodies have been identified, *Drosophila* are optimal for the in vivo analysis of the sequence and structural properties of RNA that influence phase separation. This is exemplified by studies of polar granules, a specialised population of RNP granules that are required to specify the fate of the future germ cells in a variety of metazoans [96]. In *Drosophila*, these granules share significant protein overlap with P bodies [97,98] but, critically, contain ribosomes [99] as well as polar granule specific proteins and RNAs [100]. Over 200 mRNAs are known to be enriched in polar granules [100], and those RNAs tested have been shown to be a highly stable component of the granules [101,102].

However, similarly to oocyte and embryonic P bodies, recent research has shown that it is not the RNA but the proteins that are necessary to regulate the nucleation of the polar granules [103]. Despite this, in vivo RNA, when recruited to an established granule, is capable of self-organisation into higher-order structures [104]. Transgenics and CRISPR approaches have begun to unravel how specific 3′ UTR sequences and structural components in some polar granule RNAs have the ability to drive the recruitment of the RNA to polar granules and then promote the self-assembly of these RNAs within the polar granules [102,105,106,107]. Together, this highlights the critical role that RNA, its sequence, and its structure can play in vivo to organise phase separation. Similar analyses in other condensates in *Drosophila* will reveal whether these are conserved properties of RNA.

While condensate assembly has received significant research focus, disassembly is now becoming an increasingly popular avenue of research due to the pathological implications and therapeutic potential. Again, the mature oocyte and early embryo provide an attractive in vivo system to explore the molecular mechanism of disassembly.

In the mature oocyte, P bodies have been shown to exist in a stable state, which is thought to be essential for the long-term storage of RNA and sustained translational repression [38]. The precise mechanism that leads to the disassembly of P bodies at egg activation, and the ensuing translation of the released RNA, is not well understood. In vitro studies provide insights that could be applied to articulate the pathways that regulate condensate disassembly at egg activation (Table 3).

It seems likely that these mechanisms of disassembly are interconnected in vivo. For example, we know that the calcium rise triggers changes in the post-translational modifications of P body proteins [111], which could occur through PNG, a serine-threonine kinase and the major regulator of post-translational modification at egg activation. In the unactivated oocyte, Gnu (a regulatory subunit of the PNG complex) is localised to P bodies, and, at this point in time, it is unable to bind to and activate the PNG complex [121]. It is tempting to hypothesise that the increase in cytoplasmic calcium, which leads to the activation of Gnu [111,122] and the assembly of the active PNG complex at the P body, could, in turn, disassemble the P body as a result of the phosphorylation of core P body components, the latter of which is supported by strong biochemical evidence [112].

### 4.1. Focus on: Techniques for the Study of P Bodies in Drosophila

*Drosophila* enable the combination of genetic power with a wide array of visualisation techniques for proteins and RNAs. This provides an important model system to study the biological relevance of phase separation in vivo and this subsection will cover the available techniques for this investigation.

A wealth of publicly available resources enable the tissue- or cell-specific manipulation of most genes, including their ectopic expression, overexpression, knockdown, and knockout [123,124,125]. Additionally, specific features of a protein or RNA of interest can be altered in particular cells. These methods have been successfully used to study condensates in *Drosophila*—for example, by altering protein binding sites [27,84]; adding or removing protein regions, namely known aggregation domains or intrinsically disordered regions [92]; changing post-translational modifications using phosphomimetic and non-phosphorylatable forms of a protein [92]; disrupting the RNA sequence [101,102,104]; or altering the RNA secondary structure [102,104].

**Figure 2:**
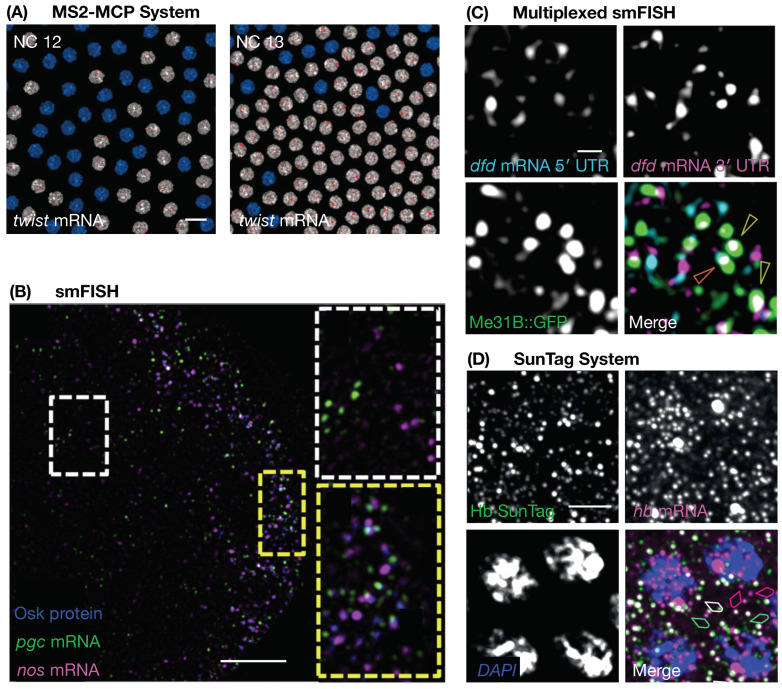
**Examples of methods to investigate RNA in *Drosophila*.** (**A**) MS2/MCP system: *twist* mRNA in nuclear cycle 12 and 13 *Drosophila* embryos, blue represents nuclei not undergoing *twist* transcription, white represent nuclei undergoing *twist* transcription and red represents a *twist* transcription foci, scale bar 10 µm, courtesy of the Lagha Lab [129]. (**B**) smFISH: *primordial germ cell (pgc)* and *nanos (nos)* mRNA visualised here simultaneously with Osk protein during late oogenesis in both the bulk cytoplasm (white box) and germ plasm (yellow box), scale bar 5 µm, courtesy of the Gavis Lab [102]. (**C**) Multiplexed smFISH: 5′ and 3′ untranslated regions (UTR) of *deformed (dfd)* mRNA visualised with Me31B protein in the early *Drosophila* embryo, orange and yellow arrow heads show co-localisation and loss of co-localisation between the 5′ and 3′ ends of the RNA respectively, scale bar 1 µm, courtesy of the Ashe Lab [54]. (**D**) SunTag system: newly translated Hunchback (Hb) protein visualised simultaneously with *hb* mRNA, magenta arrow heads show *hb* mRNA not co-localised Hb protein, green arrow heads show Hb protein not co-localised with *hb* mRNA and white arrows show a co-localisation between newly translated Hb protein and *hb* mRNA, scale bar 5 µm, courtesy of the Ashe Lab [140]. Copyright permission has been obtained for all images and there is no conflict of interest.

Advances in microscopy coupled with methods adapted specifically for *Drosophila* enable the high-resolution visualisation of the P body components and experimentation on the biological effect of altering the protein and RNA in P bodies (Figure 2 and Table 4).

Stem Loop and Coat Protein Binding Systems (MS2-MCP System): The premier method to visualise RNA in living cells is through the insertion of specific secondary structures (stem loops) into the RNA of interest (typically in the 3′ UTR). The second component, the coat protein, is conjugated to a fluorophore. When co-expressed in the same cell, the coat protein can bind to the stem loop with high affinity and thus decorate the mRNA in living cells [126]. This technique (predominantly using the MS2 bacteriophage) has been used to great success in *Drosophila* [127] and has even been optimised for single-molecule resolution at low laser power [128,129,130].

Single-Molecule Fluorescent In Situ Hybridisation (smFISH): The gold standard for the visualisation of mRNA is smFISH. By creating multiple short nucleotide oligomer probes conjugated to a fluorophore [131], it is possible to label RNA with great specificity and a minimal background. In combination with super-resolution imaging, this method can visualise the sub-granule localisation of single mRNA molecules [38,54,101,102,103,104,105,106].

Multiplexed smFISH: Two sets of smFISH probes can be created for the same RNA species: one allows for the visualisation of the 5′ UTR and the other the 3′ UTR. Full-length RNA and mRNA that have undergone 5′ to 3′ decay will have a different spectral signal, thus enabling decay intermediates to be spatially visualised [54,132]. However, RNAs with short 5′ or 3′ UTRs may be precluded from visualisation with this technique.

Translating RNA Imaging by Coat Knock-Off (TRICK): This system simultaneously utilises the MS2 and PP7 RNA stem loops and their respective coat proteins. In a single RNA species, the PP7 RNA stem loops are engineered in the open reading frame and the MS2 stem loops are placed in the 3′ UTR. When co-expressed with the coat proteins, the untranslated RNA is labelled by both coat protein fluorophores. During the pioneer round of translation, the ribosome(s) knocks off the PP7 coat protein from the RNA but the MS2 coat protein remains bound. In this way, two spectrally different signals appear from untranslated RNA and one signal from translated RNA [133,134]. This has system been successfully implemented in *Drosophila* [133,134].

SunTag System (a Novel Protein Scaffold, a Repeating Peptide Array [135]): This technique was recently adapted to *Drosophila* and allows the visualisation of the nascent translation of proteins at the single molecule level. Multiple copies of the GCN4 epitope from yeast are placed at the start of the coding sequence for a protein of interest. Once translated the epitope is recognised by a constitutively expressed cytoplasmic binding partner conjugated to a fluorophore. Multiple copies of the epitope amplify the fluorescent signal, allowing for the visualisation of single molecules of the nascent transcript in real time [129,130,135,136,137,138,139,140,141].

**Table 4:** A technical summary of the RNA and cellular process visualisation techniques.

Technique	Information Gained	Live or Fixed	Single- Molecule Resolution Feasible	Multiplexing Possible (Currently)	Signal to Noise Ratio	Potential to Affect RNA Localisation	Super- Resolution Imaging Possible	Adapted to *Drosophila*
Stem loop and coat binding system	RNA localisation [126]	Live [126]	Yes [128]	Yes [128]	Low [126]	Yes [142]	Yes [128]	Yes [127,129,130]
smFISH	RNA localisation [131]	Fixed [131]	Yes [131]	Yes [131]	High [131]	No [131]	Yes [54,101,102,103,104,105,106]	Yes [54,101,102,103,104,105,106]
Multiplexed smFISH	mRNA decay [132]	Fixed [132]	Yes [132]	Yes [132]	High [132]	No [132]	Yes [132]	Yes [54]
TRICK	Nascent translation [133,134]	Live [133,134]	Yes [133,134]	No	Low [133,134]	Yes [133,134]	Yes [133,134]	Yes [133,134]
SunTag	Nascent translation [136,137,138,139]	Live [129,136,137,138,139]	Yes [136,137,138,139]	No	High [136,137,138,139]	Low [136,137,138,139]	Yes [136,137,138,139]	Yes [129,140,141]

## 5. Using Drosophila as a Model to Understand the Role of P Bodies in Human Diseases

Combining the excellent genetic amenability with the conservation of key organ systems [143,144], *Drosophila* has a strong history in modelling a myriad of human diseases (see https://www.sdbonline.org/sites/fly/modelsystem/aamodelsystem.htm, accessed on 18 August 2023 for a comprehensive summary, or [145,146,147,148,149,150,151,152,153]). These attributes can also be applied to the study of pathogenesis caused by the dysregulation of RNP granules at a molecular, cellular, tissue, and organismal level. In *Drosophila*, most research in this area has been focused on stress granules [154,155,156,157,158] as they have more established links to disease phenotypes—for example, in amyotrophic lateral sclerosis (ALS) and frontotemporal dementia (FTD) [159,160,161,162]. However, emerging evidence suggests that P body components are likely implicated in human diseases and *Drosophila* is well equipped for research in this field.

A major constituent of Lewy bodies, a pathological hallmark of Parkinson’s disease [163], α-synuclein was recently shown to affect the modularity of P bodies in human cells and yeast [23]. Expressing human α-synuclein in *Drosophila* [164,165,166], it was confirmed that α-synuclein directly interacts with P body proteins and modulates condensate structure [23]. Moreover, the knockdown of various P body proteins in *Drosophila* modified the α-synuclein-mediated toxicity and locomotor deficiency in adult flies [23].

In patients, a rare missense mutation in DDX6 (a core P body component) leads to intellectual disability, developmental delays, and similar dysmorphic features [167]. Examined in fibroblasts, as neurones are unable to be assayed from patients, P bodies are shown to disassemble when these missense mutations are present, which leads to alterations in translation [167]. This suggests that DDX6 is likely critical for neuronal development but its role in regulating neuronal RNP granules requires additional research. Looking to the future, *Drosophila* will be a useful model to study these and other neuronal diseases [164,168].

The genetic toolkit of *Drosophila* allows the straightforward expression of patient-relevant mutations and human disease genes. Mechanistically, changes in aggregation and phase separation can be identified and connected to observable neurodegeneration phenotypes at the individual neurone and whole organism level [154,155,156,157,158,164,165,166]. The screening of genes and small molecules to identify modifiers of disease-relevant mutations is routine in *Drosophila* and may help to identify novel therapeutics [23,155,156,157,158].

Similar approaches in *Drosophila* are applicable to other complex human diseases, such as cancer, as they are well suited to experimentation on many of the hallmarks of tumorigenesis. At this time, the use of *Drosophila* in P body-specific cancer biology research is yet to be realised. What we do have evidence for shows that the link between P bodies and cancer is in its infancy, with the role of P bodies being highly dependent on the cell type and mutational history [25]. Early evidence suggests that P bodies may control changes to the translational landscape that occur during key events in disease progression, such as the epithelial to mesenchymal transition (EMT) [169,170]. *Drosophila* would be a valuable resource to test the role of P bodies in the EMT during normal development and pathogenesis.

Due to the role of P bodies in RNA metabolism, they appear to be a target for a variety of viruses, which collectively have a devastating impact on human health worldwide [171,172,173,174,175,176,177,178,179,180,181,182]. Typical P body functions are often perturbed as a consequence of viral activity in human cell lines [171], and the modulation of P bodies by viruses is conserved to *Drosophila* [172,173,174,175]. Viral proteins and RNAs can localise to and interact with P bodies, resulting in their disassembly or aggregation [173,174,175,176,177,178,179,180,181]. In addition viruses can disrupt P bodies by hijacking components for their own replication [182,183,184] or transcription [172].

A significant number of viruses that impact P bodies use arthropods as a vector—for example, the flaviviruses (Dengue virus, West Nile virus) [185], the bunyaviruses (Rift Valley Fever virus) [172,186], and the old world alphaviruses (Chikungunya virus) [187,188]. The interactions between these viruses and cellular processes may be deeply conserved as these insect-borne viruses can replicate in evolutionarily distant hosts [189]. By combining the genetic tractability and conservation of innate immune biology [190], *Drosophila* could be used to probe for the insect and human anti-viral factors that act in the interplay between P bodies and viruses.

However, the last common ancestor of humans and fruit flies lived over 600 million years ago [191] and there has been obvious divergence in this time. *Drosophila* lacks the organ system complexity of their human counterparts and data interpretation should reflect this when modelling complex diseases. Despite this, human and *Drosophila* cells are observed to be strikingly similar in both normal and diseased conditions [145]. Over 75% of human disease genes [192,193] and all known canonical human P body components have functional homologues in *Drosophila*. Thus, they are particularly well suited to understanding the fundamental mechanisms underpinning intricate human diseases.

## 6. Concluding Remarks: Drosophila as a Model for the Future Study of P Bodies

*Drosophila* is a good model to answer many outstanding questions about P bodies, RNP granules, biomolecular condensates, and phase separation in an in vivo context. This is due to the biologically relevant changes that P bodies undergo during development, the conservation of P body proteins, genetic malleability, and the high-resolution imaging techniques to examine RNA and proteins in living and fixed *Drosophila* tissue. Looking to the future, the following are major questions in the field of condensate biology that we feel *Drosophila* is particularly well suited to address:

- What regulates the assembly and disassembly of condensates in vivo?
- What effect do proteins, and their specific domains, have on condensate properties?
- What effect do post-translational modifications have on condensate integrity?
- How do RNAs, and certain motifs, contribute to the formation of RNP granules?
- Which RNA structures, sequences, and post-transcriptional modifications affect the ability of RNA to associate with RNP granules?
- Are the material properties of RNP granules intrinsic to their biological function?
- What are the biological functions of phase separation?

Time will tell whether biomolecular condensates fulfil their promise of having a major role in biology and human disease, and *Drosophila* will be important in this endeavour.

## Figures and Tables

**Table 1:** *Drosophila* P body proteins: their role, localisation to P bodies in S2 cells and/or egg chambers, and the human and budding yeast orthologues; not conserved is abbreviated to NC, (?) denotes uncertainty in the orthologue identity.

	Protein	Role	Localisation to P bodies	Human Orthologue	Yeast Orthologue
Translational regulators	eIF4E- Transporter (4E-T)	Involved in the negative regulation of the eukaryotic translation initiation factor 4F complex assembly, through competitive binding with eIF4G for eIF4E	S2 cells [39]	4E-T	NC
	Eukaryotic translation initiation factor 4E1 (eIF4E1)	Part of the eukaryotic translational initiation factor 4F complex, which is capable of binding to the 5′ mRNA cap	S2 cells and egg chamber [13,40]	eIF4E	eIF4Ep
	Maternal expression at 31B (Me31B)	A DEAD box RNA helicase that plays a critical role in translational repression and mRNA decapping	S2 cells and egg chamber [13,40]	DDX6	Dhh1p
	Trailer Hitch (Tral)	An sm-like protein with a variety of roles including translational repression and the ability to bind to DEAD box helices	S2 cells and egg chamber [13,40]	Lsm14A	Scd6p
	Staufen (Stau)	A double-stranded RNA binding protein involved in multiple mRNAs’ localisation	S2 cells and egg chamber [13,40]	Stau1	NC
Degradation machinery	Decapping protein 1 (DCP1)	A subunit of the mRNA decay holoenzyme, also involved in RNA localisation	S2 cells and egg chamber [13,14]	Dcp1	Dcp1p
	Decapping protein 2 (DCP2)	The catalytic subunit of the mRNA decay holoenzyme, an m7G(5′)pppN diphosphatase responsible for removal of the 5′ cap	S2 cells and egg chamber [13,14]	Dcp2	Dcp2p
	Enhancer of decapping 3 (Edc3)	Promotes the efficient removal of the 5′ cap from mRNA	S2 cells and egg chamber [13,14]	Edc3	Edc3p
	Ge-1	A decapping activator that couples mRNA deadenylation to decapping and may act as a scaffold to physically connect these two processes	S2 cells and egg chamber [13,41]	Edc4	NC
	Like Sm 1 (LSm1)	An sm-like protein that is part of the Lsm1-7-Pat1 complex, thought to enable RNA cap binding	S2 cells [13]	Lsm1	Lsm1p
	NOT1	Part of the CAF-1CCR4-NOT complex that degrades the mRNA poly(A) tail	S2 cells [13,14]	CNOT1	CDC39p
	Pacman (Pcm)	A 5′ to 3′ exoribonuclease that degrades decapped mRNA	S2 cells and egg chamber [13,14]	XRN1	XRN1p
	Protein associated with topo II related-1 (HPat)	A decapping activator that couples mRNA deadenylation and decapping	S2 cells and egg chamber [13,14]	Pat1A	Pat1p
	Twin (CCR4)	Part of the CAF-1CCR4-NOT complex that degrades the mRNA poly(A) tail	S2 cells [13,14]	CCR4	CCR4
miRNA machinery	Argonaute 2 (AGO2)	Interacts with small interfering RNAs (siRNAs) to form RNA-induced silencing complexes (RISCs)	S2 cells [13]	Ago2	NC
	Dicer-1 (Dcr-1)	Cleaves double-stranded RNA and is involved in the production of mature miRNAs	S2 cells [13]	Dicer-1	NC
	Dicer-2 (Dcr-2)	Cleaves double-stranded RNA and is involved in the production of mature miRNAs	S2 cells [13]	Dicer-2	NC
	Drosha	Cleaves double-stranded RNA and is involved in the production of mature miRNAs	S2 cells [13]	Drosha	NC
miRNA machinery (cont.)	Gawky (GW)	Required for gene silencing by micro-RNAs and promotes both deadenylation and decapping through the recruitment of the CCR4-NOT and the DCP1–DCP2 complexes	S2 cells [13]	GW182	NC
	Partner of Drosha (Pasha)	Cleaves double-stranded RNA and is involved in the production of mature miRNAs	S2 cells [13]	Pasha	NC
Egg chamber specific components	Bruno 1 (Bru1)	An RNA binding protein that is involved in multiple aspects of post-transcriptional gene regulation, including localisation, translational repression, and activation of translation	Egg chamber [35]	CELF1 /CELF2	WHI3p(?)
	Cup	Involved in translational repression in eIF4E dependent and independent mechanisms	Egg chamber [40]	4E-T	NC
	Exuperantia (Exu)	Involved in bcd mRNA localisation to the anterior of the oocyte in mid-oogenesis	Egg chamber [35,40,42,43]	NC	NC
	Heterogenous nuclear ribonucleoprotein at 27C (Hrb27C)	A heterogenous nuclear ribonucleoprotein, an RNA binding protein, involved in the localisation and translational regulation of mRNA	Egg chamber [40]	DAZAP1	HRP1p(?)
	Lost	Involved in mRNA localisation to the posterior of the oocyte in late oogenesis, present in multiple RNP complexes, and likely has a broader role in RNA metabolism	Egg chamber [40]	MTHFSD	Fau1p(?)
	Oo18 RNA binding protein (Orb)	Involved in mRNA polyadenylation, promoting translation (but may also act as a deadenylator and translational repressor dependent on its phosphorylation status)	Egg chamber [40]	CPEB	NC
	Squid (Sqd)	A heterogenous nuclear ribonucleoprotein A (hnRNPA), an RNA binding protein, involved in the localisation and translational regulation of grk mRNA	Egg chamber [40,42]	HNRNPAB/HNRNPD	HRP1(?)
	Ypsilon Schachtel (Yps)	RNA binding protein involved in various processes, such as translational repression and RNA stabilisation	Egg chamber [40,43]	YBX1	NC

**Table 2:** Available data on the effect that the disruption of canonical P body proteins has on the assembly and size of P bodies in five *Drosophila* tissues, human immortalised cell lines, and budding yeast. The protein used to visualise the P bodies in the experiment referenced is noted in parentheses.

	Protein Disrupted	Drosophila S2 Cells	Intestinal Stem Cells	Nurse Cells	Oocyte	Testes	Human (Adapted from [19])	Budding Yeast
Translational regulators	eIF4E- Transporter (4E-T)	-	No effect (Tral) [30]	-	-	-	Diffuse (DDX6, eIF4E, CCR4, Lsm1, Lsm14A), cannot be reinduced under stress [17,79]	Not conserved
	Eukaryotic translation initiation factor E1 (eIF4E1)	Diffuse (Me31B) [27]	No effect (Tral) [30]	-	-	-	-	-
	Maternal expression at 31B (Me31B)	Diffuse (Tral, Ge-1) [13]	Smaller (Pat1) [30]	No effect (Tral and Cup) [84]	-	-	Diffuse (Lsm1, eIF4E, CCR4, 4E-T, Edc4, Dcp1a), cannot be re-induced under stress [17,79,80]	Smaller (under starvation) (Dcp1, Dcp2, Edc3, Xrn1, Dhh1, Pat1) [85]
	Trailer Hitch (Tral)	No effect (Ge-1) [13]	Smaller (Pat1) [30]	Diffuse (Me31B) [36]	Shape altered (Me31B) [38]	-	Diffuse (Edc4, Dcp1a), cannot be re-induced under stress [78,79]	Smaller (under starvation) (Dcp2) [86]
	Staufen (Stau)	-	No effect (Tral) [30]	-	-	-	-	Not conserved
Degradation machinery	Decapping protein 1 (DCP1)	No effect (Tral, Ge1) [13]	-	Larger (Pcm) [14]	-	-	-	Larger (unstressed) (Ccr4, Dhh1, Pat1, Lsm1, Xrn1, Dcp2, Edc3) [85]
	Decapping protein 2 (DCP2)	Larger (Tral, Ge1) [13]	No effect (Tral) [30]	Larger (Dcp1) [14]	-	-	Larger (LSm1, DDX6, eIF4E, CCR4) [17] No effect (Ge-1) [87]	Smaller (under starvation) (Ccr4, Dhh1, Pat1, Lsm1, Xrn1, Edc3) [85]
	Enhancer of decapping 3 (Edc3)	No effect (Tral, Ge1) [13]	Larger (Tral) [30]	-	-	-	No effect (Edc4) [79]	Smaller (under starvation) (Dhh1, Pat1, Lsm1, Dcp1, Dcp2, Xrn1) [83]
Degradation machinery (cont.)	Ge-1	Diffuse (Tral) [13]	Diffuse (Tral) [30]	-	Diffuse [41]	-	Smaller/fewer/diffuse, can be re-induced by stress (DDX6, Lsm14A, Dcp1a) [79,87]	Not conserved
	Like Sm 1 (LSm1)	Diffuse (Tral, Ge1) [13]	Larger (Tral) [30]	-	-	-	Diffuse (DDX6, eIF4E, CCR4, 4E-T) [17]	Larger (unstressed) (Dcp1, Dcp2, Edc3, Xrn1, Dhh1, Pat1) [85]
	Not1	Diffuse (Tral, Ge1) [13]	Diffuse (Tral) [30]	Not localised to P bodies [14]	-	-	-	-
	Pacman (Pcm)	Larger (Tral, Ge1) [13]	Larger (Tral) [30]	Larger (Dcp1, Dcp2) [14]	-	Larger (Dcp1) [31]	Larger (Dcp2) [16]	Larger (unstressed) (Ccr4, Dhh1, Pat1, Lsm1, Dcp1, Dcp2, Edc3) [85]
	Protein associated with topo II related-1 (HPat)	Diffuse (Tral, Ge1) [13]	Diffuse (Tral) [30]	-	-	-	Smaller/fewer/diffuse, can be re-induced by stress (Edc4) [76,88,89]	Smaller (Dcp1, Dcp2, Edc3, Xrn1, Dhh1, Pat1) [85]
	Twin (CCR4)	-	No effect (Tral) [30]	Not localised to P bodies [14]	-	-	Diffuse (DDX6, eIF4E, Lsm1, 4E-T) [17]	Smaller (under starvation) (Dcp2, Edc3, Dhh1, Pat1, Lsm1, Xrn1, Dcp1) [85]
miRNA machinery	Argonaute 2 (AGO2)	Diffuse (Tral, Ge1) [13]	No effect (Tral) [30]	-	-	-	-	Not conserved
	Dicer-1 (Dcr-1)	Diffuse (Tral, Ge1) [13]	No effect (Tral) [30]	-	-	-	-	Not conserved
	Dicer-2 (Dcr-2)	Diffuse (Tral, Ge1) [13]	No effect (Tral) [30]	-	-	-	-	Not conserved
	Drosha	Diffuse (Tral, Ge1) [13]	No effect (Tral) [30]	-	-	-	-	Not conserved
	Gawky (GW)	Diffuse (Tral, Ge1) [13]	No effect (Tral) [30]	-	-	-	Diffuse, can be re-induced by stress (Dcp1a, Lsm4) [80,90]	Not conserved
	Partner of Drosha (Pasha)	Diffuse (Tral, Ge1) [13]	No effect (Tral) [30]	-	-	-	-	Not conserved

**Table 3:** A summary of the in vitro mechanisms of the disassembly of condensates and the parallel mechanisms that occur at egg activation.

In Vitro Mechanisms for Disassembling Condensates	Comparative Mechanisms for Disassembling Condensates at Egg Activation
Changes in the ionic concentration [108]	An increase in the intracellular calcium level [53,109]
Changes to post-translational modifications [110]	Phosphorylation of P body components [111,112]
Changes to the protein concentration [108,110]	Swelling and increase in volume [113,114,115,116,117], lowering of the cytoplasmic concentrations of P body proteins
Changes to the cytoskeletal architecture [118,119]	Multiple instances of actin cytoskeleton remodelling [120]

## Data Availability

Data sharing not applicable.
